# The effects of sarcopenic dysphagia on the dynamics of swallowing organs observed on videofluoroscopic swallowing studies

**DOI:** 10.1111/joor.12936

**Published:** 2020-02-09

**Authors:** Taishi Miyashita, Takeshi Kikutani, Keigo Nagashima, Kumi Igarashi, Fumiyo Tamura

**Affiliations:** ^1^ Division of Clinical Oral Rehabilitation The Nippon Dental University School of Life Dentistry at Tokyo Tokyo Japan; ^2^ Department of Rehabilitation for Speech and Swallowing Disorders Tama Oral Rehabilitation Clinic The Nippon Dental University School of Life Dentistry at Tokyo Koganei Japan

**Keywords:** larynx, pharyngeal muscles, sarcopenia, swallowing

## Abstract

**Objectives:**

The aim of the present study was (a) to determine the relationship of videofluoroscopic swallowing study (VFSS) findings of the swallowing musculature with the diagnostic criteria for sarcopenic dysphagia and (b) to examine the usefulness of VFSS for diagnosing sarcopenic dysphagia.

**Methods:**

The participants were 132 patients (mean age, 80.4 ± 8.8 years). Their skeletal muscle mass, nutritional status and swallowing functions as assessed by VFSS findings were measured. Also, the relationship between the VFSS findings and sarcopenia was examined.

**Results:**

Of all the participants, 20 men (mean age, 83.2 ± 6.9 years) and 27 women (mean age, 85.3 ± 6.9 years) were diagnosed with sarcopenia. In men, the amount of laryngeal upward movement (ALUM) was significantly lower and the pharyngeal area was significantly wider in the sarcopenia group than in the non‐sarcopenia group. In women, the pharyngeal area was significantly wider in the sarcopenia group than in the non‐sarcopenia group. In a logistic regression model, ALUM (odds ratio [OR] 1.135, 95% confidence interval [CI] 1.037‐1.241, *P* = .006) and pharyngeal area (OR 0.028, 95% CI 0.001‐0.670, *P* = .027) was a significant independent factor for the presence or absence of sarcopenia.

**Conclusions:**

The decline in swallowing function of sarcopenia patients was characterised by lower laryngeal movement and enlargement of the pharyngeal cavity due to decreased skeletal muscle mass and decreased muscle strength. The present study suggested the usefulness of measuring ALUM during swallowing and measuring the pharyngeal area with VFSS as indicators of decreased swallowing muscle function in sarcopenia.

## 1 INTRODUCTION

Sarcopenia, which occurs with ageing, disrupts the vital functions of older people. Some people with decreased systemic muscle mass show a decline in swallowing function, a concept described as sarcopenic dysphagia.^1^ When a patient presents clinically with dysphagia without a distinct underlying illness such as cerebrovascular disease, the cause of dysphagia could be sarcopenia. The concept of sarcopenic dysphagia is considered to be “dysphagia due to sarcopenia of the whole body and swallowing muscles, and includes as a cause secondary sarcopenia due to age, decreased activity, malnutrition and disease (invasive and cachexia).”^2^ Sarcopenia is a serious cause of dysphagia elders. Sarcopenic dysphagia is often observed after aspiration pneumonia.^3^ The treatment for aspiration pneumonia often requires patients to have nothing ingested by mouth and complete bed rest, which results in aggravation of sarcopenia^2^ during the pneumonia treatment and may increase the severity of dysphagia. The relationship between aspiration pneumonia, one of the most common causes of death in older people, and sarcopenic dysphagia implies that sarcopenia is ultimately linked to a fatal outcome.

Recently, diagnostic criteria for sarcopenic dysphagia were proposed.^2^ According to this proposal, a prerequisite for diagnosing sarcopenic dysphagia is a decrease in systemic muscle mass. Further criteria include declines in muscle mass and muscle strength of the swallowing muscles, as well as the presence or absence of decreased physical function. MRI measurements obtained by Molfenter et al showed increased pharyngeal area due to pharyngeal muscle atrophy in ageing.^4^ Enlargement of the pharyngeal cavity then leads to pharyngeal contractile dysfunction during swallowing, causing pharyngeal residue after swallowing food, which in turn increases the risk for aspiration.^5^ Steele et al found that reduced movement of the hyoid‐larynx complex increased the risk of laryngeal penetration and aspiration.^6^ These findings suggested that sarcopenia due to ageing may affect muscle strength and muscle mass of the pharyngeal muscles, leading to decreased swallowing function. If the presence of sarcopenic dysphagia can be identified based on the findings of the gold standard of swallowing function examinations, such as the videofluoroscopic swallowing study (VFSS), we may be able to show that sarcopenia is the cause of dysphagia.

The aim of the present study was (a) to determine the relationship of VFSS findings of the swallowing organs with the diagnostic criteria for sarcopenic dysphagia and (b) to examine the usefulness of VFSS for diagnosing sarcopenic dysphagia.

## 2 MATERIALS AND METHODS

### 2.1 Participants

The participants were 132 patients (mean age, 80.4 ± 8.8 years) who visited our clinic with a complaint of swallowing disorders due to choking at meals and difficulty in eating between January 2017 and May 2018. They were tested by VFSS as part of their swallowing test; patients with neuromuscular disease, oropharyngeal cancer and cerebrovascular disease with marked paralysis, as well as those who could not follow instructions, were excluded. There were 73 men (mean age, 80.3 ± 8.2 years) and 59 women (mean age, 80.5 ± 9.7 years).

### 2.2 Measured parameters

Sex, age, primary disease and Barthel index date (BI)^7^ were collected as basic information. Nutritional status was evaluated using the Mini Nutritional Assessment‐Short Form (MNA‐SF)^8^ and body mass index as indicators, and sarcopenia‐related parameters were assessed using the skeletal muscle mass index (SMI) and hand grip strength. The Food Intake Level Scale (FILS) was used to evaluate feeding status.^9^

The bioelectrical impedance analysis method (InBody S10, Model JMW140; BioSpace) was used to measure SMI. Muscle mass of the extremities (the sum of the muscle mass of the upper and lower limbs) divided by height (m) squared was the SMI value.^10^ Hand grip strength was measured using a hand grip dynamometer (TKK5401 Grip‐D; Smedley, Takei Scientific Instruments). Measurements were taken twice from both the left and right sides. The highest measurement was recorded as the hand grip strength.^10^ The participants were divided into two groups, the sarcopenia group and the non‐sarcopenia group. The diagnosis of sarcopenia was made based on the diagnostic criteria of sarcopenia^10^ proposed by the Asian Working Group for Sarcopenia. The participants were diagnosed as having sarcopenia when both the hand grip strength (<26 kg in men and <18 kg in women) and SMI (≤7.0 kg/m^2^ in men and <5.7 kg/m^2^ in women) were below the criteria levels.

For the VFSS, participants were asked to sit upright at 90° with the neck in the neutral position. The patients were asked to slowly exhale completely (maximal expiratory level). This was considered the resting state. The laryngeal position at rest (LPR) and pharyngeal area were measured from the images taken at rest. Next, the participants were asked to swallow 3 ml of thickened liquid (nectar‐like),^11^ which was placed sublingually with a syringe by the examiner. To examine the dynamics of each dynamic aspect of swallow organ during swallowing, the amount of laryngeal forward and upward movements was measured from the images taken during swallowing.

**Figure 1:**
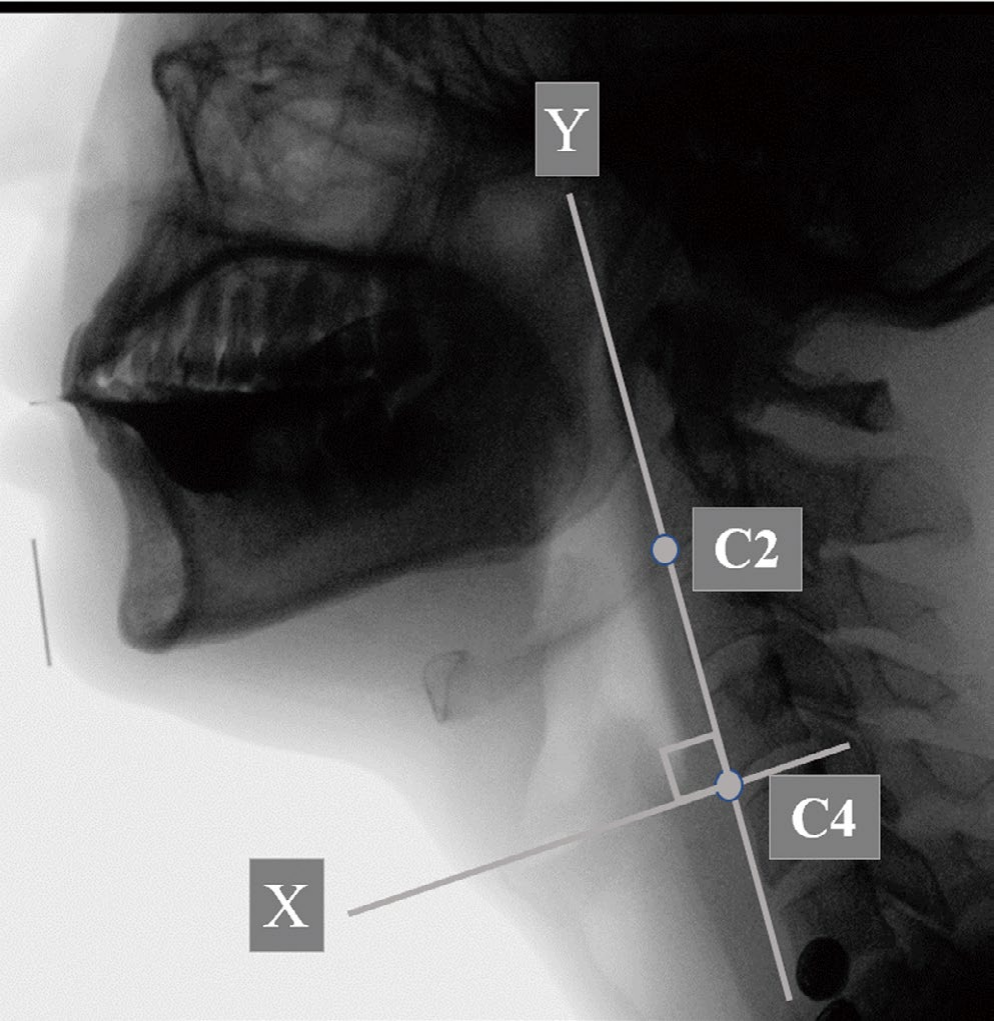
Setting of the reference axes and pharyngeal area outlined in the lateral view. C4, anterior ridge of fourth cervical vertebra, defined as the origin of the coordinate axis; C2, anterior ridge of second cervical vertebra; y‐axis, the line passing through both C2 and C4; x‐axis, the line perpendicular to the y‐axis passing through the origin (C4); hyoid position, X‐Y coordinate point (x, y), relative to the origin

A coordinate system with the y‐axis forming a straight line connecting the lower ends of the second and fourth cervical vertebrae and the x‐axis forming a straight line drawn perpendicular from the lower end of the fourth cervical vertebra to the y‐axis was established. The lower end of the fourth cervical vertebra was established as the point of origin, and the upper end of the body of the thyroid cartilage was established as the position of the thyroid cartilage. The distance from the point of origin to the X‐Y coordinates (mm) was then measured.^12^ The length was corrected using the metal marker of 20.0 mm in diameter as a reference for the measurement of images (Figure 1).

Each of the parameters was measured using the following measurement methods. To measure LPR, two lines perpendicular to the y‐axis, one through a point on the anterior inferior margin of the second cervical vertebra and the other through the bottom point of the epiglottic vallecula, were drawn. LPR was the distance between the two intersection points.

**Figure 2:**
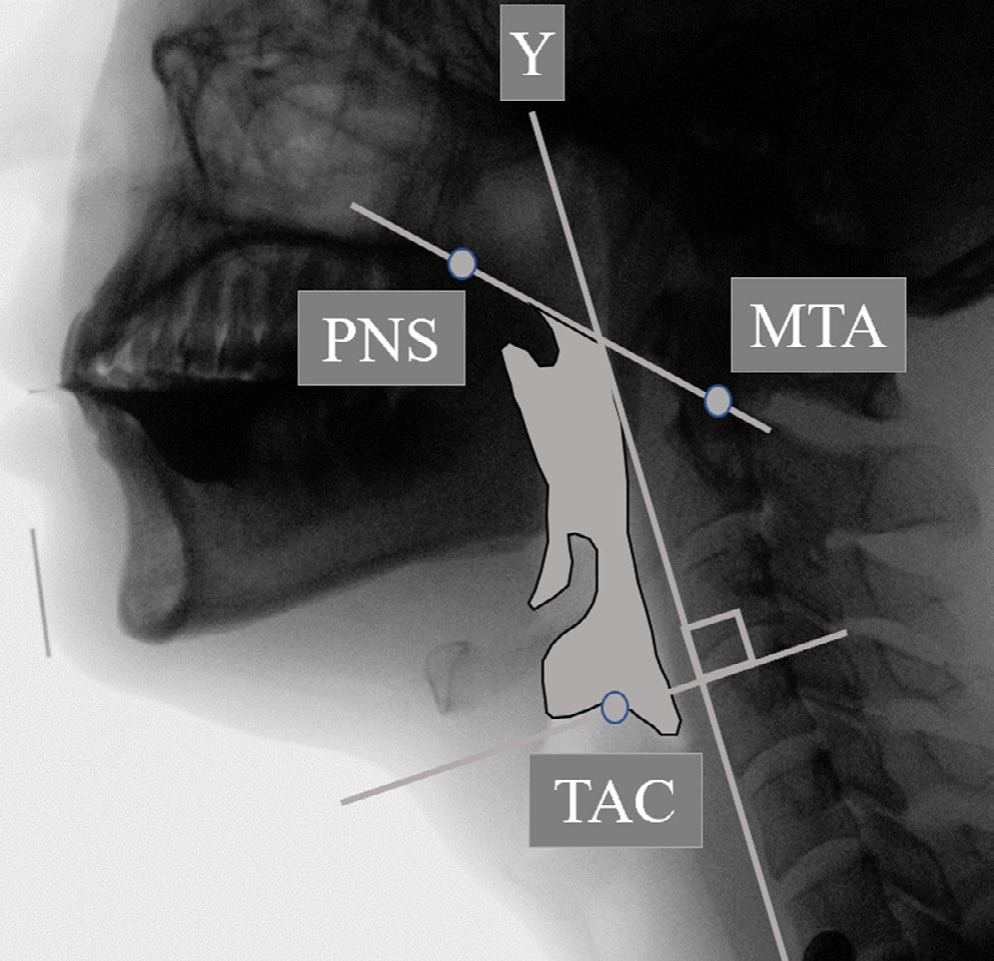
Outline of the pharynx area. The image shows a tracing of the pharyngeal area in grey. PNS, the posterior nasal spine; MTA, the midportion of the tubercle of the atlas; TAC, the top of the arytenoid cartilages

The outline of the pharyngeal area was defined posteriorly by the posterior pharyngeal wall from the midportion of the tubercle of the atlas down to the level corresponding to the height of the top of the arytenoid cartilages. The inferior outline was carried forward from this point over the arytenoid cartilages and anteriorly to outline the epiglottis, vallecula and the tongue base to the point at which the soft palate came into contact with the tongue base. The outline was then carried over the pharyngeal surface of the soft palate to the point of contact with the nasal spine. The superior border was a straight line between the posterior nasal spine and the midpoint of the tubercle (Figure 2). To correct for any differences in pharyngeal size across participants, the pharyngeal area measurements were normalised using the squared length of the C2‐C4 vertebral distance.^5^, ^13^

To measure laryngeal movement, markers were placed on the anterior superior margin of the thyroid cartilage and the anterior inferior margins of the second and fourth cervical vertebrae. Using the level at rest prior to starting the swallowing motion, the movement paths of the coordinate points were recorded. The movement paths were separated into vectors along the x‐axis and y‐axis, and they were measured as the amount of laryngeal forward movement (ALFM) and the amount of laryngeal upward movement (ALUM).

The measurements were made using image analysis software (ImageJ; National Institutes of Health) and two‐dimensional motion analysis software (DIPP‐Motion V/2D ®; DITECT).

### 2.3 Statistical methods

The differences in basic information, nutritional status, sarcopenia‐related parameters and VFSS parameters between the sarcopenia group and the non‐sarcopenia group were tested using the *t* test. A multiple logistic regression model was used to identify factors that were independently associated with sarcopenia. The participants with non‐sarcopenia group served as a reference.

Statistical analysis was performed using the IBM SPSS® Ver24.0 statistical analysis software (IBM SPSS Japan). The significance level was set at 5%.

## 3 RESULTS

The feeding functions of the 132 participants were as follows: 30 participants (15 men and 15 women) were at FILS Level 7 (“Orally ingesting three meals consisting of easy‐to‐swallow foods. No nutritional support is needed”); 51 participants (29 men and 22 women) were at FILS Level 8 (“Orally ingesting three meals, except foods that are especially difficult to swallow”); and 51 participants (29 men and 22 women) were at FILS Level 9 (“No dietary restriction. Orally ingesting three meals”).

There was significant sex difference in the SMI (*P* < .001) and hand grip strength (*P* < .001). They were significantly higher in men. Of the VFSS parameters, ALUM (*P* < .001) and LPR (*P* = .001) were significantly higher in men. ALFM (*P* = .007) was significantly higher in women (Table 1).

**Table 1:** Background characteristics of the study participants

	All (N = 132)	Men (N = 73)	Women (N = 59)	*P*‐value
	Mean ± SD	Mean ± SD	Mean ± SD	
Age (year)	80.4 ± 8.8	80.3 ± 8.2	80.5 ± 9.7	.890
BI	87.42 ± 20.73	90.07 ± 16.47	84.15 ± 24.78	.119
MNA‐SF	10.15 ± 2.71	10.42 ± 2.91	9.81 ± 2.44	.200
BMI (kg/m^2^)	20.59 ± 3.62	20.97 ± 3.28	20.11 ± 3.99	.180
SMI	6.15 ± 1.08	6.80 ± 0.85	5.34 ± 0.72	<.001
Hand grip strength (kg)	23.56 ± 9.45	28.52 ± 9.06	17.41 ± 5.52	<.001
ALFM (mm)	9.01 ± 3.33	8.31 ± 3.52	9.88 ± 2.88	.007
ALUM (mm)	32.92 ± 8.3	36.78 ± 7.62	28.15 ± 6.44	<.001
Pharyngeal area (%)	0.70 ± 0.19	0.72 ± 0.20	0.67 ± 0.17	.175
LPR (mm)	23.57 ± 9.11	25.98 ± 8.30	20.59 ± 9.25	.001

Abbreviations: ALFM, amount of laryngeal forward movement; ALUM, amount of laryngeal upward movement; BI, Barthel index; BMI, body mass index; LPR, laryngeal position at rest; MNA‐SF, Mini Nutritional Assessment‐Short Form; SD, standard deviation; SMI, skeletal muscle mass index.

A total of 47 participants (20 men with a mean age of 83.2 ± 6.9 years and 27 women with a mean age of 85.3 ± 6.9 years) were diagnosed with sarcopenia according to the sarcopenia diagnostic criteria, and 85 participants (53 men with a mean age of 79.2 ± 8.4 years and 32 women with a mean age of 76.3 ± 10.0 years) were not diagnosed with sarcopenia.

The differences in each measured parameter between the sarcopenia and non‐sarcopenia groups were examined. In men, BI (*P* < .001), MNA‐SF (*P* < .001), BMI (*P* < .001), SMI (*P* < .001), hand grip strength (*P* < .001) and ALUM (*P* = .015) were significantly lower in the sarcopenia group. Age (*P* = .045), and the pharyngeal area (*P* = .002) was significantly wider in the sarcopenia group than in the non‐sarcopenia group. In women, BI (*P* = .002), MNA‐SF (*P* < .001), SMI (*P* < .001) and hand grip strength (*P* < .001) were significantly lower in the sarcopenia group. Age (*P* < .001) and the pharyngeal area (*P* = .002) were significantly higher in the sarcopenia group than in the non‐sarcopenia group (Table 2).

**Table 2:** Difference in sarcopenia group and non‐sarcopenia group in each measurement item

	All		*P*‐value	Men		*P*‐value	Women		*P*‐value
	Sarcopenia group (N = 47)	Non‐sarcopenia group (N = 85)		Sarcopenia group (N = 20)	Non‐sarcopenia group (N = 53)		Sarcopenia group (N = 27)	Non‐sarcopenia group (N = 32)	
	Mean ± SD	Mean ± SD		Mean ± SD	Mean ± SD		Mean ± SD	Mean ± SD	
Age (year)	84.4 ± 6.9	78.0 ± 9.1	<.001	83.2 ± 6.9	79.2 ± 8.4	.045	85.3 ± 6.9	76.3 ± 10.0	<.001
BI	75.52 ± 26.80	94.43 ± 11.77	<.001	76.25 ± 19.32	95.19 ± 11.84	<.001	75.00 ± 31.42	93.29 ± 11.75	.002
MNA‐SF	8.44 ± 2.28	11.15 ± 2.42	<.001	8.00 ± 2.36	11.34 ± 2.56	<.001	8.75 ± 2.20	10.86 ± 2.21	<.001
BMI (kg/m^2^)	18.90 ± 3.72	21.51 ± 3.16	<.001	18.23 ± 2.92	21.96 ± 2.82	<.001	19.36 ± 4.16	20.82 ± 3.57	.147
SMI	5.30 ± 0.78	6.61 ± 0.93	<.001	5.94 ± 0.64	7.13 ± 0.68	<.001	4.83 ± 0.49	5.76 ± 0.60	<.001
Hand grip strength (kg)	15.51 ± 5.00	28.00 ± 8.33	<.001	18.83 ± 4.89	32.18 ± 7.43	<.001	13.06 ± 3.46	21.08 ± 4.06	<.001
ALFM (mm)	8.77 ± 3.37	9.14 ± 3.33	.546	8.23 ± 3.94	8.34 ± 3.39	.907	9.17 ± 2.89	10.47 ± 2.78	.085
ALUM (mm)	29.96 ± 7.57	34.56 ± 8.27	.002	33.29 ± 7.65	38.10 ± 7.26	.015	27.49 ± 6.61	28.70 ± 6.35	.477
Pharyngeal area (%)	0.78 ± 0.21	0.65 ± 0.16	<.001	0.83 ± 0.26	0.67 ± 0.16	.002	0.74 ± 0.16	0.61 ± 0.15	.002
LPR (mm)	24.79 ± 7.94	22.90 ± 9.67	.253	27.06 ± 5.92	25.57 ± 9.05	.500	23.12 ± 8.89	18.46 ± 9.13	.053

Abbreviations: ALFM, amount of laryngeal forward movement; ALUM, amount of laryngeal upward movement; BI, Barthel index; BMI, body mass index; LPR, laryngeal position at rest; MNA‐SF, Mini Nutritional Assessment‐Short Form; SD, standard deviation; SMI, skeletal muscle mass index.

We carried out logistic regression analysis with basic information and VFSS parameters as independent variables, and the presence or absence of sarcopenia as the dependent variable. The analysis showed that BI (odds ratio [OR] 1.047, 95% confidence interval [CI] 1.011‐1.084, *P* = .009), MNA‐SF (OR 1.436, 95% CI 1.085‐1.900, *P* = .011), ALUM (OR 1.135, 95% CI 1.037‐1.241, *P* = .006) and pharyngeal area (OR 0.028, 95% CI 0.001‐0.670, *P* = .027) were significantly related to the presence or absence of sarcopenia (Table 3).

**Table 3:** Logistic regression analysis for factors related to outcomes for people with or without sarcopenia

Independent variables	B	SE	Wald	P	OR	95% CI	
						Lower limit	Upper limit
Sex	−0.076	0.629	0.014	.904	0.927	0.270	3.181
Age	−0.052	0.035	2.177	.140	0.949	0.886	1.017
BMI	0.144	0.090	2.570	.109	1.155	0.968	1.378
BI	0.046	0.018	6.776	.009	1.047	1.011	1.084
MNA‐SF	0.362	0.143	6.402	.011	1.436	1.085	1.900
ALUM	0.126	0.046	7.612	.006	1.135	1.037	1.241
Pharyngeal area	−3.589	1.627	4.868	.027	0.028	0.001	0.670

Abbreviations: ALUM, amount of laryngeal upward movement; BI, Barthel index; BMI, body mass index; CI, confidence interval; MNA‐SF, Mini Nutritional Assessment‐Short Form; OR, odds ratio; SE, standard error; SMI, skeletal muscle mass index

## 4 DISCUSSION

In both men and women, hand grip strength, SMI and ALUM were significantly lower in the sarcopenia group, and pharyngeal area was significantly wider in the sarcopenia group. These findings showed that sarcopenia, with decreased skeletal muscle mass and muscle strength as its principal signs,^14^, ^15^ affects not only a decrease in systemic skeletal muscle mass, but also the muscle mass of swallowing muscles and the amount of laryngeal movement during swallowing.

In this study, LPR was shown to be higher in women than in men. Furthermore, sarcopenia did not affect laryngeal elevation in women. Previous reports have shown that in older people, the position of the larynx relative to the skull is relatively lower than in younger people.^16^ The larynx position at rest is said to differ between men and women,^17^ and similar results were obtained in this study. Inamoto et al found that there was a significant gender difference in the hyoid bone position and laryngeal size when examining the pharynx and larynx in adults.^18^ From these things, it was thought that the larynx that remained high was not easily affected by sarcopenia during swallowing.

The enlargement of the pharyngeal cavity is associated with pharyngeal contractile dysfunction during swallowing. In addition, pharyngeal contractile dysfunction is related to pharyngeal residue after swallowing, which increases the risk of aspiration.^5^ Furthermore, the decrease in the amount of movement of the hyoid‐larynx complex has been shown to result in increased risk of laryngeal penetration and aspiration.^6^ The reason may be that decreased movement of the hyoid‐larynx complex leads to epiglottic inversion dysfunction, and the resulting compromised epiglottic closure increases the risk of aspiration. The above findings suggest that sarcopenia is involved in enlargement of the pharyngeal cavity and reduced laryngeal elevation, and it may become a serious problem by reducing swallowing function and increasing the risk of aspiration.

When the pharyngeal area was examined by sex, in both men and women, pharyngeal area was significantly wider in the sarcopenia group; however, low laryngeal elevation was only observed in men in the sarcopenia group. With sarcopenia, there may be a difference in how the effects on swallowing muscles are manifested. The decrease with age in muscle strength represented by hand grip strength and knee extension strength is known to be more notable in men.^19^ In addition, there are studies that have shown the same for tongue pressure^20^ and biting force^21^ of the swallowing muscles. In recent years, intramuscular adipose tissue, which is present in the muscles, is attracting attention as an indicator of sarcopenia. Deposition of intramuscular adipose tissue has been suggested to reduce muscle strength.^22^ Intramuscular adipose tissue has also been shown to be very closely associated with age in men.^23^ Compared to women, ageing seems to cause changes in not only muscle mass, but also changes in intramuscular quality in men in particular. According to Akima et al, in both men and women, there was a significant negative correlation between intramuscular adipose tissue and muscle thickness. Similarly, there was a negative correlation between intramuscular adipose tissue and muscle strength to perform motor functions.^23^ These findings suggest that the presence of sarcopenia is not only related to a wider pharyngeal area, an indication of reduced muscle mass, but in men, it also strongly affects laryngeal elevation related to muscle strength.

The results of this study showed that sarcopenia was associated with pharyngeal area and LPR. This result indicates that it is important to determine whether the cause of dysphagia is associated with sarcopenia. Once the sarcopenic dysphagia diagnostic criteria on VFSS are established, prompt diagnosis becomes possible. This will be useful in the rehabilitation of patients with sarcopenic dysphagia.

The present study has some limitations. First, each of the measurements obtained on VFSS may not be the absolute representative value of the patient. The reason is due to the difficulty in strictly regulating the posture of the patients during VFSS. In the present study, an attempt was made to standardise the posture of the patients as much as possible. Second, the data collected in the present study were based on the measurement of one swallowing movement. Normally, data are collected multiple times, and attempts are made to homogenise the data. In consideration of the risk of radiation exposure from VFSS imaging, only limited numbers of imaging sessions necessary to obtain a clinical diagnosis were performed to minimise participants’ radiation exposure.

## 5 CONCLUSION

The sarcopenia group showed lower ALUM and wider pharyngeal area than the non‐sarcopenia group. The measurement of ALUM and pharyngeal area by the VFSS may be useful as an index to indicate the functional deterioration of swallowing muscles due to sarcopenia.

## CONFLICT OF INTEREST

The authors declare no conflict of interest.

## AUTHOR CONTRIBUTIONS

Indicate authors’ role in study concept and design, acquisition of subjects and/or data, analysis and interpretation of data, and preparation of manuscript. Takeshi Kikutani conceived and designed the study, conducted the study, acquired subjects and data, interpreted the data and prepared the manuscript. Taishi Miyashita acquired the subjects and data, performed statistical analysis, interpreted the data and prepared the manuscript. Keigo Nagashima acquired the subjects and data, interpreted the data and prepared the manuscript. Kumi Igarashi and Fumiyo Tamura interpreted the data and prepared the manuscript.
